# TME11/385: International Pathology Consultations through Internet E-mail and High Resolution Digital Photomicrography

**DOI:** 10.2196/jmir.1.suppl1.e118

**Published:** 1999-09-19

**Authors:** V Della Mea, L Mariuzzi, CA Beltrami, A Marchevsky

**Affiliations:** ^1^University of Udine, UdineItaly

**Keywords:** Telemedicine, Remote Consultation, Telepathology, Internet, Electronic Mail

## Abstract

**Introduction:**

Remote consultation though static telepathology, i.e. based on the delivery of still histology images, is one of the most interesting as well as discussed telemedicine fields, due to its suitability for an email implementation, and to the problem of the preliminary image sampling. Current scanning digital cameras can acquire digital images at very high resolutions that allow the acquisition of microscopic images at low optical magnification, decreasing the number of photographs necessary to represent a histology slide. A small number of images can be thus tiled to represent an entire biopsy, obtaining a visual representation that resembles that of light microscope. In this way, no real sampling is needed. The present paper discusses this approach in conjunction with the Internet Electronic mail.

**Methods:**

The acquisition device: the images were acquired through a photoscanner, which incorporates a linear CCD chip, that slides onto the image field as in flatbed scanners. This allows for very high resolutions (up to 3400x2700 pixels) at a cost considerably lower than equivalent digital cameras, but with higher time for acquisition (1-2 minutes). This allows for the acquisition of low magnification large fields which at the same time contain as much detail as higher magnification fields. 20:1 JPEG compression has been adopted for reducing storage size. E-mail protocols and formats: MIME is a message format for heterogeneous, possibly encrypted and signed, multimedia e-mail still deliverable by the usual protocols, i.e. SMTP and POP. MIME is adequate for representing a static telepathology case composed of text data and a set of histology images. In our study, we used the Netscape Communicator 4.0 mail agent. Material: The study included two steps. The former involved the delivery of 31 cases acquired by a senior pathology resident, the latter 30 cases acquired by a physician with little pathology knowledge, both in Cedars-Sinai Medical Center and e-mailed to Udine University for interpretation.

**Results:**

In the first step, twenty-eight cases (93.5%) were correctly diagnosed by telepathology. In the second step, two cases had insufficient images, and seventeen (60.7%) were correctly diagnosed by telepathology. Experiments in sending digitally signed and encrypted cases have been done, with some interoperability problem.

**Discussion:**

High-resolution digital photomicrography and the Internet offer useful tools for telepathology consultations. Our preliminary results show that the diagnostic accuracy decreases with the knowledge of the image sampler. This makes static telepathology more suitable for remote expert consultation than as a mean for primary diagnosis on material selected by a technician. Furthermore, this technology is probably best suited for the study of small specimens, such as biopsies. However, e-mail protocols provide for a range of features that, when used, makes its use highly valuable in telemedicine and telepathology.

